# Tobacco Cessation Interventions in Dental Networks: A Practice-based Evaluation of the Impact of Education on Provider Knowledge, Referrals, and Pharmacotherapy Use

**Published:** 2006-06-15

**Authors:** Nicolaas P Pronk, Darla Havlicek, Eric Stafne

**Affiliations:** HealthPartners Health Behavior Group; HealthPartners Health Behavior Group, Minneapolis, Minn; Tobacco Cessation Clinic, University of Minnesota School of Dentistry, Minneapolis, Minn

## Abstract

Tobacco is a significant risk factor for oral diseases. Dental care providers have the opportunity to inform patients about the risks associated with tobacco use and refer them to tobacco cessation resources. Although dental teams usually ask their patients about their tobacco use, most do not provide tobacco cessation counseling.

This project involved four staff-model dental clinics and four contracted network dental clinics. Project goals were to 1) describe current practice patterns of tobacco cessation intervention, 2) increase the use of steps for treatment, known as the *5 As*, recommended by the U.S. Public Health Service, 3) increase referrals to a tobacco helpline, and 4) increase use of pharmacotherapy for tobacco dependence treatment. The project included training and program support (e.g., sharing of project data, weekly newsletters, discussion at clinic meetings). Results indicate that this approach to addressing tobacco dependence in a dental clinic setting can effectively change dental provider knowledge and action.

## Introduction

Tobacco use is one of the most significant causative and contributing factors for oral cancers and periodontal diseases (1) and contributes to altered wound healing and less successful oral disease treatment results (2). In its position statement on tobacco, the American Dental Association urges its members "to become fully informed about tobacco cessation intervention techniques to effectively educate their patients to overcome their addiction to tobacco" (3). Dental clinics emphasize prevention of oral diseases, educate patients about oral health, and treat patients on a recurring basis. The dental office is therefore an ideal setting to encourage and assist patients to become tobacco-free.

The U.S. Public Health Service clinical practice guideline for treating tobacco use and dependence in a health care setting recommends a treatment model involving the following five steps, called the *5 As*: 1) ask about tobacco use, 2) advise to quit, 3) assess willingness to make a quit attempt, 4) assist in quit attempt, and 5) arrange for follow-up (4). The 5 As are widely accepted as the standard for tobacco cessation interventions (4).

Literature reviews show that tobacco cessation interventions are not a routine part of dental practice (5-9). This project was designed to address tobacco dependence among patients in dental practices by educating dental care providers about tobacco cessation and providing them with supportive programming. Project goals were to 1) describe current practice patterns of tobacco cessation interventions, 2) increase the use of the 5 As, 3) increase referrals to a tobacco cessation helpline, and 4) increase pharmacotherapy for treating tobacco dependence. The impact of this project was evaluated over the course of 1 year among dental care providers in the HealthPartners health insurance plan. HealthPartners is a not-for-profit, consumer-governed, integrated health care system providing medical and dental health care benefits and care delivery services to a membership of approximately 650,000 in the Upper Midwest.

## Project Overview

The dental division of HealthPartners includes 16 staff-model clinics in the HealthPartners Dental Group and 60 contracted dental networks in Minnesota. The project was implemented in four of the staff-model clinics and four network clinics from Park Dental, which, with 26 clinics in Minneapolis, is the largest of the contracted networks. Participating clinics were selected based on their interest. A steering committee, which included clinic staff, as well as training and data committees guided the project. Project components included 1) training dental office teams in tobacco cessation interventions; 2) providing follow-up support, including weekly electronic newsletters, sharing of project data, and attendance at clinic staff meetings to discuss project implementation; and 3) collecting and managing data to monitor the project's effectiveness. HealthPartners conducted provider training and provided resources, including access to two tobacco cessation telephone-based programs. The HealthPartners phone-based tobacco cessation program (for HealthPartners members only) included up to nine outbound calls per member in addition to a structured curriculum and self-help materials. The Minnesota Tobacco Helpline (for non-HealthPartners members) included four outbound calls. Both telephone-based programs were provided free of charge and focused on topics such as planning for quitting, being prepared for withdrawal challenges, preventing relapse, using quit-smoking medications, and managing weight. Staff from HealthPartners' Center for Health Promotion coordinated the project.

## Training

Training of the dental office teams was led by a practicing dentist with academic credentials and consisted of two 2-hour sessions per clinic site. Sixty-six staff members from the HealthPartners Dental Group clinics and 79 dental team members from the Park Dental clinics received the training in November 2001. Participants included dentists, registered dental hygienists, dental assistants, receptionists, and office managers. Staff hired after the training began attended an abbreviated 2-hour training session.

Before the training began, a baseline survey was administered to dentists, registered dental hygienists, and dental assistants in the entire HealthPartners Dental Group system and the four Park Dental project sites. The survey assessed knowledge and practices of behavior change and the 5 As, documentation of patients' tobacco status, and prescriptions for pharmacotherapy. Surveys were repeated 1 year after the training.

The first training session addressed why dental teams should be interested and involved in helping patients with tobacco cessation. Information was provided on risks of tobacco use and oral health, benefits of a tobacco-free lifestyle, nicotine addiction, the 5 As, and pharmacotherapy. In addition, the transtheoretical model of behavioral change was described (10). The session also addressed how the counseling process fits into the clinic's work flow. Routine and brief interventions that would not disrupt clinic operations were emphasized. To increase documentation of tobacco status and use of the 5 As, chart stickers and forms were developed. Staff members were encouraged to use the 5 As stickers to document their interactions with patients. Stickers were attached to the progress notes section of the patient's chart. For patients who set a tobacco quit date, call reminder cards and follow-up chart stickers were used. 

Several codes were used to identify tobacco users. The American Dental Association code (D1320) and the HealthPartners tobacco cessation intervention code (I1321) were used by HealthPartners Dental Group clinics. The tobacco cessation intervention code (0007) was used by Park Dental practices. Codes were defined as "tobacco counseling for the control and prevention of oral disease."

To help make dental teams more comfortable addressing tobacco use with patients, scenarios and a list of commonly asked questions were developed for the training. Scenarios were introduced using role-play techniques.

The second training session focused on the role of dentists in prescribing pharmacotherapy and provided information about various products (nicotine patch, gum, lozenge, inhaler, spray, and bupropion). Tobacco cessation resources and information on how to access these resources were provided. Dental teams were asked to refer patients interested in quitting to either the HealthPartners phone-based tobacco cessation program or the statewide Minnesota Tobacco Helpline.

## Project Support

Project components designed to support clinics in their efforts to successfully address tobacco dependence were offered throughout the project. Data were shared with participating clinic staff on coding, referrals to and enrollment in the HealthPartners phone-based tobacco cessation program, and pharmacy prescriptions. Tobacco-related information was shared through weekly e-mailed newsletters that included "hot tips" on tobacco cessation. Project staff met regularly with clinic staff to answer questions, discuss concerns, and provide updates on new cessation resources. This supportive programming was designed to keep the project visible and maintain the effects of the training.

## Measures

Several evaluation components were incorporated into the project. These included baseline and 12-month posttraining surveys and chart reviews to monitor documentation of the 5 As, use of dental codes and referral resources, pharmacy prescriptions written and filled, and referral to and enrollment in the HealthPartners phone-based tobacco cessation program.

We compared the four HealthPartners Dental Group pilot clinics with the other 12 HealthPartners Dental Group clinics (control group) not participating in the project. This comparison allowed us to consider the impact of training on prescription rates and fill patterns for tobacco cessation pharmacotherapy through the HealthPartners Dental Group. We focused the analysis on how effectively the training addressed tobacco dependence. A pre–post single group design was used for both clinic groups, and chi-square analyses were conducted to test for differences in proportions. Statistical significance was set at α = .05.

At the end of the project, we reviewed implementation of activities with project coordinators from both dental groups. The purpose was to learn qualitatively which dynamics were considered important in program implementation. Issues such as leadership, project management, technical difficulties, and clinical processes were addressed.

## Results

Baseline survey results showed that knowledge and use of the 5 As and behavioral change theory were low in both clinic groups. Posttraining results indicated significant improvement in both the HealthPartners Dental Group and Park Dental clinic system in knowledge and practices of behavior change according to the transtheoretical model and the 5 As of tobacco cessation. Significant improvements in 5 As awareness were found among both clinic systems, which resulted in increased rates of asking, advising, assessing, assisting, and arranging for follow-up. Whereas not all of the 5 As actions increased significantly, all increased in the desired direction (Table 1).

**Figure 1:**
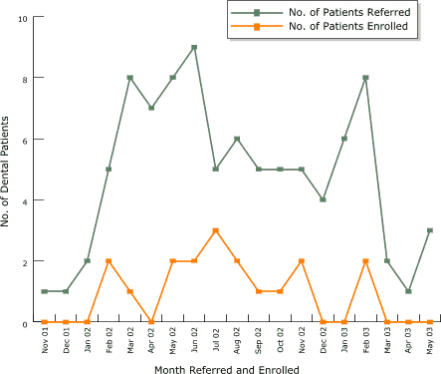
Number of HealthPartners Dental Group members within the four intervention clinics referred by dental staff and enrolled in the HealthPartners tobacco cessation telephone course from November 2001 to June 2003 as measured by telephone helpline data. Dental staff training was held in November 2001.

Throughout the project, we kept track on a monthly basis of HealthPartners Dental Group members who were referred to and enrolled in the HealthPartners phone-based tobacco cessation program (Figure 1). During the project, the process of applying the 5 As resulted in increased patient referrals to phone-based tobacco cessation counseling. Of the 93 patients referred by their providers to phone-based counseling during the project, 17 (18%) enrolled.

**Table:** 

**Figure 2:**
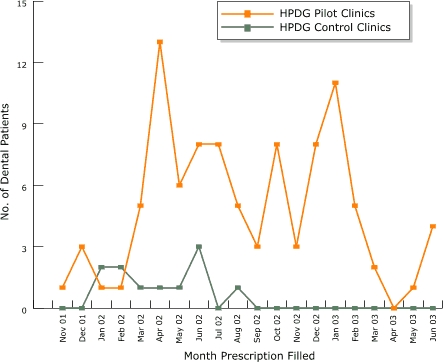
Number of dental patients at the four HealthPartners Dental Group (HPDG) pilot clinics and 12 nonpilot clinics (control group) with a prescription written and filled for nicotine replacement therapy or bupropion from November 2001 to June 2003, as measured by pharmacy data. Dental staff training was held in November 2001.

Pharmacy data were reviewed and tracked monthly to determine the number of HealthPartners members who filled pharmacotherapy prescriptions. We compared results between the four HealthPartners Dental Group pilot clinics and the 12 HealthPartners Dental Group nonpilot clinics (control group). Results presented in Figure 2 indicate an increase in filled prescriptions for pharmacotherapy compared with HealthPartners Dental Clinics that did not receive the intervention.

**Table:** 

Survey results showed that dental staff documented tobacco status in various sections of the patients' dental charts (Table 2). At baseline, tobacco status was most often documented in the health history forms and periodontal risk assessment sections of the HealthPartners Dental Group chart. The progress notes and dental examination charting sections also had frequent documentation of tobacco status. However, only the remainder of the chart not covered by these four sections (designated as *other*) showed significant improvement at posttraining. Overall, the documentation rate among the staff-model clinics improved from 84% to 88%.

Within the Park Dental clinic group, tobacco status was more likely to be documented in the progress notes at baseline (Table 2). The posttraining survey results for this group showed a significant increase in documentation of tobacco status using the health history form as well as the other section. Overall, the documentation rate increased from 83% to 91% for the Park Dental group.

The qualitative data from the project review sessions revealed several important lessons about factors in the project's success. First, involvement of clinic leaders was crucial to maintaining the project's momentum. The monthly newsletters and data also helped sustain participants' interest and remind them of project goals. Coordinators and staff felt ownership of the process because of an up-front agreement on project goals, objectives, and implementation processes. For example, time constraints for clinic staff in addressing tobacco use with patients was a concern at every clinic. Finally, clearly defined roles and responsibilities related to the project were very important.

From the final review of the project with the dental groups, we identified several factors that allowed the work to flow smoothly. First, training included all members of the dental office team: dentists, dental hygienists, dental assistants, receptionists, and office managers. Buy-in by the whole team was critical to successful integration of tobacco cessation interventions within the clinic setting. Next, periodic follow-up with clinics was important to keep the project visible to clinic staff. The quarterly meetings and weekly electronic newsletters provided information to dental staff on clinic performance that was well received and seemed to generate an ongoing connection with the program and feeling of ownership.

## Discussion

Our project demonstrated that with systematic training and posttraining follow-up support, routine tobacco cessation practices can be documented, and dental patients can be assisted efficiently in their attempts to be tobacco-free. Therefore, we have concluded that training implemented in a real-world clinic setting can be an effective strategy for addressing tobacco dependence.

Whereas the data derived from surveys, chart reviews, and pharmacy records showed significant improvements, the project review sessions provided us with insights about factors that contributed to the project's success. Leadership involvement and support, the feeling of ownership among those involved in the implementation, and the need to make the process a successful part of daily clinic operations were identified as the most important factors to sustaining the project after training.

Finally, the project had some limitations. First, the project was implemented among clinics that expressed an interest and a willingness to participate. In addition, the clinics enrolled in the project belonged to only two dental groups in a single geographic region. These factors may limit the generalizability of the intervention.

## Conclusion

This project documented increased prescriptions for nicotine replacement products and bupropion, increased referrals to and enrollment in telephone-based counseling courses, increased knowledge and practices of behavior change, and increased use of the 5 As in response to a systematically implemented training program enhanced with supportive programming.

Leadership involvement, agreement on project processes, agreement on participants' responsibility and accountability, clearly defined roles, and a means of dealing effectively with time constraints appear to be critical factors to successful implementation.

## Figures and Tables

**Table 1:** Pretraining and Posttraining Knowledge and Practices of Behavior Change and the 5 As^a^ for Tobacco Cessation Among Dental Care Providers in Eight Clinics

**Knowledge or Practice**	**Provider Group**					

	**HealthPartners Dental Group (Staff-Model Clinics)**			**Park Dental (Contracted Network)**		

	**Baseline, %(n = 205)**	**Posttraining, %(n = 118)**	** *P* Value^b^**	**Baseline, %(n = 64)**	**Posttraining, %(n = 33)**	** *P* Value^b^ **
Knowledge of transtheoretical model	12	37	<.001	6	58	<.001
Knowledge of 5 As	3	35	<.001	6	64	<.001
Ask rate	45	59	.01	27	33	.49
Advise rate	49	61	.03	44	67	.03
Assess rate	32	49	.002	25	52	.009
Assist rate	22	30	.10	3	46	<.001
Arrange rate	7	12	.13	3	39	<.001

a The 5 As are steps in tobacco cessation interventions recommended by the U.S. Public Health Service: 1) ask about tobacco use, 2) advise to quit, 3) assess willingness to make a quit attempt, 4) assist in quit attempt, and 5) arrange for follow-up (4).

b Significance of differences determined by chi-square test.

**Table 2:** Results of Pretraining and Posttraining Survey of Dental Care Providers About Documentation of Patient Tobacco Status^a^

**Section of Patient Chart**	**Provider Group**					

	**HealthPartners Dental Group (Staff-Model Clinics)**			**Park Dental (Contracted Network)**		

	**Baseline, %(n = 205)**	**Posttraining, %(n = 118)**	***P* Value^b^**	**Baseline, %(n = 64)**	**Posttraining, %(n = 33)**	** *P* Value^b^**
Health history form	61	65	.51	38	64	.02
Progress notes	36	44	.18	64	76	.24
Dental examination charting	13	13	.90	3	3	.98
Periodontal risk assessment	59	64	.38	5	9	.39
Other^c^	1	12	<.001	0	9	.01
Overall documentation rate	84	88	.36	83	91	.21

a Percentages do not necessarily add up to 100% because a patient's chart may include documentation on tobacco use in more than one section.

b Significance of differences determined by chi-square test.

c Last section of patient chart for additional information.
